# Sequencing and analysis of the complete mitochondrial genome of large-toothed Siberian shrew (*Sorex daphaenodon*)

**DOI:** 10.1080/23802359.2019.1666039

**Published:** 2019-09-16

**Authors:** Xingyao Chen, Bo Pang, Dajie Xu, Xiufeng Yang, Honghai Zhang, Huashan Dou

**Affiliations:** a Hulunbuir Academy of Inland Lakes in Northern Cold & Arid Areas, Hulunbuir, P. R. China;; b College of Life Science, Qufu Normal University, Qufu, P. R. China

**Keywords:** *Sorex daphaenodon*, mitochondrial genome, phylogenetic analysis

## Abstract

The mitochondrial genome of *Sorex daphaenodon* was sequenced and analyzed for the first time using muscle tissue. This genome was 17351 bp in length and contained 13 protein-coding genes, 22 tRNA genes, and 2 rRNA genes, 1 control region and 1 rep_origin. The phylogenetic analysis basis of 12 protein-coding genes except for ND6 gene of 13 species shows that *Sorex daphaenodon* close with *Sorex tundrensis*, and was farthest related to *Sorex cylindricauda* in the genus of *Sorex*.

The *Sorex daphaenodon* is classified under order Eulipotyphla, family Soricidae and genus *Sorex*. This species has a large population size and distributed from Ural mountains to Pacific Ocean included China, Russian Federation and Mongolia. It’s diet consists of earthworms, spiders, millipedes, and insects (Sokolov et al. 1985; Chotolchu and Stubbe 2008). In this study, the tissue sample of *S. minutissimus* was catched by pitfall traps though field survey, and the geo-spatial coordinates are 48°22′19″N latitude and 117°31′54″E longitude. The sample was store in the Animal Specimen Museum of Qufu Normal University, Qufu, Shandong, China and the accession number is QFA20180063. All sampling procedures and experimental manipulations held the proper permits. After manual annotated and assembled, this mitochondrial genome was submitted in GeneBank with the accession number MK641806.

This mitochondrial genome was 17,351 bp in length and contained 13 protein-coding genes, 22 tRNA genes, 2 rRNA genes, 1 control region, and 1 rep_origin. Among these genes, nine genes (*ND6* and eight tRNA) encoded in L-strand and other genes encoded in H-strand. The percent of base composition is 33.2% for A, 28.9% for T, 13.1% for G, 24.8% for C, and the A and T (62.1%) is higher than G and C (37.9%). The gene arrangement and content are similar to the complete mitochondrial genome of other mammal species (Huang et al. 2016; Yang et al. 2019).

Phylogenetic analysis of 13 species (include 9 *Sorex* species, 2 *Episoriculus* species, and 2 *Blarinella* species) were analyzed using the Bayesian inference (BI) and maximum likelihood (ML) methods based on the 12 protein-coding genes except *ND6* with *M. arvalis* used as outgroup. The mode of GTR + I + G was selected as the best-fitting nucleotide substitution mode according to the AIC criterion by MrModeltest 3.7 (Nylander 2004). BI and ML analysis with a bootstrap test of 100 relicates by MrBayes 3.2.2 (Ronquist and Huelsenbeck 2003) and PAUP 4.0b10 (Swofford 2002) was run for 1,000,000 generations used this mode, respectively.

The result of phylogenetic analysis shows that the different methods (ML and BI) have the same topology structure with strong support for all nodes (Figure 1). It shows that two major phyletic lineages were present in Soricidae. The first group includes *Episoriculus* and *Blarinella*, the other nine *Sorex* species make up the second group. *Sorex daphaenodon* was close to *S. tundrensis* and was farthest related to *S. cylindricauda* which was diverged first in the genus of *Sorex*. Additionally, *S. tundrensis* and *S. cylindricauda* showed close relationship in our phylogenetic analysis, which was also supported by previous studies (Xu et al. 2015). We expect the present study to provide a useful database for further research and phylogenetic relationship of Soricidae.

**Figure 1.**
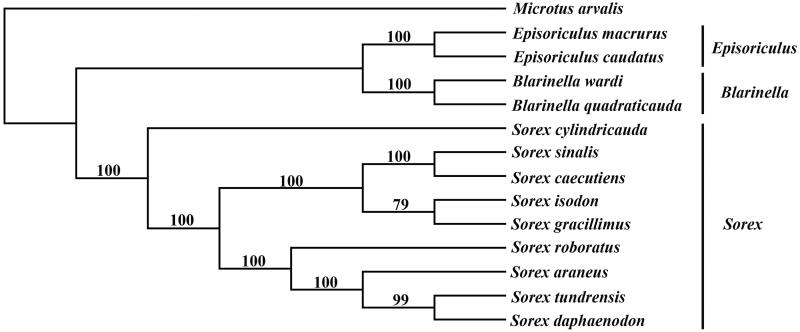
Phylogenetic tree using maximum-likelihood (ML) and Bayesian phylogenetic inference (BI) method based on 12 protein-coding genes except *ND6* of 13 species that belong to the genus of *Sorex*, *Episoriculus* and *Blarinella* and the BI posterior probabilities are shown on the nodes. The species accession numbers were downloaded from GenBank are *E. macrurus* (NC_029840), *E. caudatus* (NC_026131), *B. wardi* (MF125692), *B. quadraticauda* (NC_023950), *S. cylindricauda* (KT023074), *S. sinalis* (NC_037174), *S. caecutiens* (MF374796), *S. isodon* (NC_037894), *S. gracillimus* (NC_037859), *S. roboratus* (NC_034808), *S. araneus* (NC_027963), *S. tundrensis* (NC_025327) and *M. arvalis* (NC_038176), respectively.
